# A human leukocyte antigen imputation study uncovers possible genetic interplay between gut inflammatory processes and autism spectrum disorders

**DOI:** 10.1038/s41398-023-02550-y

**Published:** 2023-07-06

**Authors:** Laura Lombardi, Sigrid Le Clerc, Ching-Lien Wu, Jihène Bouassida, Wahid Boukouaci, Sobika Sugusabesan, Jean-Romain Richard, Mohamed Lajnef, Maxime Tison, Philippe Le Corvoisier, Caroline Barau, Tobias Banaschewski, Rosemary Holt, Sarah Durston, Antonio M. Persico, Bethany Oakley, Eva Loth, Jan Buitelaar, Declan Murphy, Marion Leboyer, Jean-François Zagury, Ryad Tamouza

**Affiliations:** 1 Université Paris Est Créteil, INSERM U955, IMRB, Laboratoire Neuro-Psychiatrie translationnelle, F-94010 Créteil, France; 2 Laboratoire Génomique, Bio-informatique et Chimie Moléculaire (EA7528), Conservatoire National des Arts et Métiers, 292, rue Saint Martin, 75003 Paris, France; 3 HESAM Université, Paris, France; 4 Université Paris Est Créteil, Inserm, Centre Investigation Clinique, CIC 1430, Henri Mondor, Créteil, F94010 France; 5 Plateforme de Ressources Biologiques, HU Henri Mondor, Créteil, F94010 France; 6 Child and Adolescent Psychiatry and Psychotherapy, Central Institute of Mental Health, Medical Faculty Mannheim, University of Heidelberg, Mannheim, Germany; 7 Autism Research Centre, Department of Psychiatry, University of Cambridge, Cambridge, UK; 8 Education Center, University Medical Center Utrecht, Utrecht, The Netherlands; 9 Child and Adolescent Neuropsychiatry Program at Modena University Hospital, & Department of Biomedical, Metabolic and Neural Sciences, University of Modena and Reggio Emilia, Modena, Italy; 10 Department of Forensic and Neurodevelopemental Science, Institute of Psychatry, Psychology and Neuroscience, King’s College London, London, United Kingdom; 11 Department of Cognitive Neuroscience, Donders Institute for Brain, Cognition and Behaviour, Nijmegen, The Netherlands; 12 Université Paris Est Créteil, INSERM U955, IMRB, Laboratoire Neuro-Psychiatrie translationnelle, AP-HP, Hôpital Henri Mondor, Département Médico-Universitaire de Psychiatrie et d’Addictologie (DMU IMPACT), Fédération Hospitalo-Universitaire de Médecine de Précision (FHU ADAPT) and Fondation FondaMental, Créteil, F-94010 France

**Keywords:** Personalized medicine, Diagnostic markers

## Abstract

Autism spectrum disorders (ASD) are neurodevelopmental conditions that are for subsets of individuals, underpinned by dysregulated immune processes, including inflammation, autoimmunity, and dysbiosis. Consequently, the major histocompatibility complex (MHC)-hosted human leukocyte antigen (HLA) has been implicated in ASD risk, although seldom investigated. By utilizing a GWAS performed by the EU-AIMS consortium (LEAP cohort), we compared HLA and MHC genetic variants, single nucleotide polymorphisms (SNP), and haplotypes in ASD individuals, versus typically developing controls. We uncovered six SNPs, namely rs9268528, rs9268542, rs9268556, rs14004, rs9268557, and rs8084 that crossed the Bonferroni threshold, which form the underpinnings of 3 independent genetic pathways/blocks that differentially associate with ASD. Block 1 (*rs9268528-G*, *rs9268542-G*, *rs9268556-C,* and *rs14004-A*) afforded protection against ASD development, whilst the two remaining blocks, namely *rs9268557-T,* and *rs8084-A*, associated with heightened risk. rs8084 and rs14004 mapped to the *HLA‐DRA* gene, whilst the four other SNPs located in the *BTNL2* locus. Different combinations amongst *BTNL2* SNPs and HLA amino acid variants or classical alleles were found either to afford protection from or contribute to ASD risk, indicating a genetic interplay between *BTNL2* and HLA. Interestingly, the detected variants had transcriptional and/or quantitative traits loci implications. As BTNL2 modulates gastrointestinal homeostasis and the identified HLA alleles regulate the gastrointestinal tract in celiac disease, it is proposed that the data on ASD risk may be linked to genetically regulated gut inflammatory processes. These findings might have implications for the prevention and treatment of ASD, via the targeting of gut-related processes.

## Introduction

Autism spectrum disorders (ASD) are complex and heterogeneous neurodevelopmental conditions characterized by problems in social interaction and verbal and non-verbal communication, repetitive behavior, and stereotyped behaviors and interests [1]. Along with co-occurring psychiatric symptoms, patients with ASD also present with several medical comorbidities including seizures, sleep disorders, and gastrointestinal dysfunctions, particularly with alterations in bowel habits and abdominal pain evident in 20–90% of ASD patients [2]. Various genetic and environmental factors associated with ASD have an impact on brain development [3, 4] and/or on the homeostasis of immune responses [5]. Environmental risks not only include prenatal factors such as maternal obesity, valproate use, and infections [6], but also gut microbial commensals dysregulation [7, 8]. Growing evidence in recent decades has highlighted the role of alterations in immune function, including heightened inflammation, anti-brain protein antibodies, as well as changes in T-cell and natural killer (NK) cell function in individuals diagnosed with ASD [5, 9, 10].

Immune dysregulation in ASD is primarily comprised of (i) an apparent increased susceptibility to prenatal infectious, possibly leading to maternal immune activation with consequent deleterious neurodevelopmental processes [11–13]; (ii) a pro-inflammatory status observed long after disease onset and possibly resulting from non-resolved infectious events [14, 15]; (iii) phenotype and functional alterations in immune cell subsets [16]; (iv) inflammation-mediated gut alterations [17–19]; and (v) increased ‘autoimmune’ disorders in ASD patients and their mothers, sometimes in association with raised circulating anti-brain protein autoantibodies [15, 20].

As with many other common immune-related disorders, these immune tags are believed to stem from complex interactions between inter-individual genetic backgrounds and environmental insults. Exploring the basis of their immunogenetic control is of interest not only for the understanding of the underlying immune processes but also for the discovery of predictive, diagnostic, and prognostic biomarkers that may help in the identification of homogeneous sub-groups of ASD patients, and thereby to preventative and symptom-targeted therapies.

One of the most promising immune genetic candidates is the human leucocyte antigen (HLA) system, which is hosted by the major histocompatibility complex (MHC) on the short arm of chromosome 6 (6p21.3). HLA genes encode cell surface molecules pivotal for cellular and humoral adaptive immune responses. The HLA/MHC cluster recently joined the psychiatric at-risk genomic regions either at pan-genomic or at a case-control level. More precisely, (i) a meta-analysis of genome-wide association studies (GWAS) performed in different psychiatric disorders, including ASD, detected the MHC region as a strong signal of risk [21], (ii) a more recent and targeted post-GWAS HLA imputation allowed to identify a protective effect conferred by an HLA-DPB1 variant against autism and intellectual disability [22] and (iii) we reported both at risk and protective HLA haplotypes associated with ASD through HLA haplotype-based analysis [23–25]. In the latter context, the uncovered risk modulator haplotypes are highly implicated in inflammatory processes during chronic inflammation and ‘autoimmunity’, including gut dysbiosis [25–28]. Moreover, HLA molecules are pivotal for central nervous system (CNS) development and function, including core neuronal functions such as neuronal/synaptic plasticity, pruning, learning, memory, and behavior [29–32], as well as more direct modulation of neuron-neuron interactions and neuro-signaling [33].

Despite the functional importance of the HLA system in patterned immune responses, it remains understudied in psychiatric and neurodevelopmental disorders, including ASD. This seems partly due to the high levels of polymorphisms that characterize the HLA loci, coupled with the complexity of the MHC-hosting HLA complex, which is one of the most gene-dense regions of the human genome [34]. The HLA gene cluster is physically divided into three functionally distinct sub-regions namely: (i) the HLA-class I region includes the classical *HLA-A, HLA-B*, and *HLA-C* genes as well as the non-classical *HLA-E*, *HLA-F* and *HLA-G* loci. These HLA genes differentially regulate antigen presentation to CD8^+^ T lymphocytes towards adaptive cellular immune responses, and immunomodulatory properties important for immune surveillance and inflammation homeostasis; (ii) the HLA class II region that is mainly comprised of *HLA-DPA1*, *HLA-DPB1*, *HLA-DQA1*, *HLA-DQB1*, *HLA-DRA*, *HLA-DRB1*, *HLA-DRB3*, *HLA-DRB4* and *HLA-DRB5* genes, which are involved in the presentation of antigens to CD4^+^ T cells and the initiation of adaptive humoral immune responses; and (iii) the class III region which contain gene loci involved in inflammatory responses, leukocyte maturation and in the complement cascade (for review see Tamouza et al., 2021 [25]).

HLA-class I and class II genes are also referred to as histocompatibility loci. These encode cell surface expressed molecules crucial to self or foreign antigen presentation to T-cell receptors (TCR) on effector cells. HLA-class I and class II genes also underpin the downstream modulation of adaptive immune responses, with their dysfunctions relevant to anti-infectious responses, inflammatory processes, ‘autoimmunity’ and/or intestinal dysbiosis [35]. The complexity of HLA loci is indicated by the presence of more than 35,000 alleles reported to date [36] and by their association with a range of diverse medical conditions, including inflammatory/’autoimmune’, cancers and, more recently, psychiatric disorders [37, 38]. HLA loci are therefore important in unraveling the complexity of pathophysiological processes across a range of diverse medical conditions. The high level of linkage disequilibrium present in the HLA/MHC region, together with the strong homogeneity of HLA histocompatibility loci, requires expensive and time-consuming high-resolution sequencing approaches as well as a high degree of expertize in the assignment of allelic polymorphisms and the association signals. Alternatively, imputation tools of the HLA diversity from GWAS chip data are now available and provide accurate information regarding single nucleotide polymorphisms (SNPs), alleles, genotypes, haplotypes, and amino-acids position of the implicated loci within the MHC region [39].

Accordingly, we took advantage of a GWAS performed by a large European consortium named “EU-AIMS” (A Multicentre Study for Developing New Medications)” to perform an in-depth analysis of the MHC/HLA genetic variants possibly influencing ASD risk and/or severity. We specifically investigated the distribution of HLA and MHC-imputed genetic variants corresponding to amino acid, SNP alleles, and haplotypes in ASD individuals, versus typically developing controls. Importantly, we narrowed the ASD phenotype by including in the analysis only patients of European descent with a strict diagnosis of ASD without intellectual disability and unrelated non-affected controls. We believed that applying such stringent selection would allow us to uncover variants otherwise difficult to be detected because of potential dilution effects.

## Materials and methods

### Longitudinal European Autism Project (LEAP): design, participant selection, and clinical characterization

The present study was performed under the framework of the “European Autism Interventions—A Multicentre Study for Developing New Medications (EU-AIMS)” project. Funded by the European community, EU-AIMS was designed to identify ASD markers to enhance earlier and more accurate diagnosis and prognosis as well as the development of new therapies. More specifically the EU-AIMS Longitudinal European Autism Project (LEAP) aims to identify risk factors that contribute to differences in brain development, difficulties in social behavior, and other core ASD symptoms. Consequently, an accelerated longitudinal design was used. Four sub-cohorts of ASD individuals and non-autistic controls, defined by age and intellectual quotient (IQ), were recruited concurrently i.e. (i) adults aged 18–30 years; (ii) adolescents aged 12–17 years; (iii) children aged 6–11 years with IQ in the typical range (75+); and (iv) adolescents and adults aged 12–30 years with mild intellectual disability (ID) (IQ 50–74). This approach allowed the investigation of the age range of interest in a shorter time period as compared to a single-cohort approach. The protocol and standard operation procedures (SOPs) of the LEAP study are accessible on https://www.eu-aims.eu/fileadmin/websites/eu-aims/ media/EU-AIMS_LEAP/EU-AIMS-LEAP_SOP_Study- Protocol.zip. The study was approved by national and local ethics review boards at each study site and is carried out under Good Clinical Practice (ICH GCP) standards.

Patients enrollment criteria were as follows: (i) a clinical diagnosis of ASD based on DSM-IV/ICD-10 [40, 41] or DSM-5 criteria [1], (ii) age between 6 and 30 years, (iii) confirmation of the ASD diagnosis using the Autism Diagnostic Interview-Revised (ADI-R) [42] and the Autism Diagnostic Observation Schedule 2 (ADOS-2) [43], (iv) eligibility of participants with psychiatric comorbidities with exception for psychosis or bipolar disorder, (v) eligibility of participants under stable medication. Assessment of symptom severity across core domains of ASD was conducted, with emphasis on social communication, repetitive and restricted behaviors, and sensory processing anomalies. Exclusion criteria consisted of: (i) significant hearing or visual impairments not corrected by glasses or hearing aids, (ii) history of alcohol and/or substance abuse or dependence within the year preceding the date of inclusion, (iii) presence of any MRI contraindications, and (iv) individuals with low IQ (<50) due to the difficulty of administering core measures and MRI scanning without sedation.

For the typically developing (TD) group, beside strict exclusion for any personal or familial history of psychiatric disorder, criteria used for the ASD group were applied. Additionally, TD individuals were required to have a T-score below 70 on the social responsiveness scale [44], a psychometric tool used to identify and quantify social impairment associated with ASD.

Inclusion and exclusion criteria for ASD patients as well as for the typically developing (TD) group were as described previously [45, 46].

Participants were recruited between January 2014 and March 2017 across six European specialized ASD centers namely the: (i) Institute of Psychiatry, Psychology and Neuroscience, King’s College London (IoPPN/KCL, UK), (ii) Autism Research Centre, University of Cambridge (UCAM, UK), (iii) University Medical Centre Utrecht (UMCU, Netherlands), (iv) Radboud University Medical Centre (RUMC, Netherlands), (v) Central Institute of Mental Health (CIMH, Germany) and (vi) the University Campus Bio-Medico (UCBM) in Rome, Italy. In addition, twins discordant with ASD were recruited at the Karolinska Institute in Sweden. A variety of recruitment sources were utilized, including existing volunteer databases, existing research cohorts, clinical referrals from local outpatient centers, special needs schools, mainstream schools, and local communities.

The study sample of the LEAP cohort is comprised of genetic data on 1566 individuals from 698 families, including 51 from twin families.

For the present investigation, we designed a case-control study including ASD patients (without intellectual disability) and controls belonging to the TD group. Eligible ASD cases were all participants who have an IQ greater than 75 without any exclusion for missing clinical data. Parents and relatives were excluded from the analysis even if classed as having ASD. Moreover, given the well-known restricted anthropogenic distribution of HLA diversity, only subjects of European descents were kept in the analysis.

### Genotyping procedure and quality controls

Genetic data from the LEAP cohort participants and their families were obtained for 1566 individuals (coming from 698 families, including 51 twin families). Their genotyping was performed at the “Centre National de Recherche en Génomique Humaine” (CNRGH) using the Infinium OmniExpress-24v1 BeadChip (721114 markers) from Illumina.

We performed a genetic quality control on the whole genetic LEAP cohort and the following SNPs were removed: 41,889 with missingness rate >2%, 17,832 from sexual chromosomes, 61,701 with minor allele frequency <1% and 459 with departure from Hardy–Weinberg equilibrium (HWE) with *p* value < 10^−6^ for controls and *p* value < 10^−10^ for cases. For individual quality control of the 1566 ASD participants, 28 individuals were removed when data missingness rate per individual >2%, and 3 individuals removed with sex discrepancies, with no individuals removed for a heterozygosity rate issue. Finally, we obtained 599,233 SNPs for 1535 individuals.

### Phenotype

Given the case-control design of our study, further exclusions were made from the 1535 individuals that passed the genetic quality control procedure, including all the parents remaining in the cohort (*N* = 818), even when diagnosed with ASD. In the remaining individuals (*N* = 717), we identified as cases, all LEAP individuals that passed the genetic quality control with an ASD diagnosis and with IQ > 75 (*N* = 328). Included controls were all LEAP individuals that passed the genetic quality control and classed according to the TD criteria (*N* = 282). This procedure of selection was used in order to narrow the well-known heterogeneity of ASD even if we have to mention that participants with IQs between 75 and 85 may exhibit some intellectual impairments. Moreover, we excluded 16 cases and 38 controls due to sibling relationships. Finally, 556 individuals, including 312 cases and 244 controls survived remained. We performed a principal component analysis (PCA) on genotype data of the 556 selected individuals, leading to 28 individuals being excluded due to non-European ethnic origins to avoid spurious associations. At the end, 298 ASD participants without ID and 230 unrelated controls, all of European descents were retained for further statistical analysis. The flow chart of the participants selection procedure is depicted in supplemental Fig. S1.

To evaluate the potential impact of the genetic diversity on ASD severity, we used the following psychometric tools from among those utilized in the LEAP clinical evaluation: (i) the Autism Diagnostic Interview (ADI) [42], with its 3 domains—social, restricted and repetitive behaviors (rrb) and communication difficulties; (ii) the Autism Diagnostic Observation Schedule (ADOS) [43] Calibrated Severity Scores (CSS) total, social affect and rrb; (iii) the SSP (Short Sensory Profile) [47] total score for quantifying sensory processing disorder in ASD patients; (iv) the Strength and Difficulties Questionnaire (SDQ) externalizing score [48]; (v) the Social Responsiveness Scale [44] T-score, as a quantitative phenotype of autistic traits; (vi) DAWBA scores [49] for depression, anxiety, Attention Deficit—Hyperactivity disorders (ADHD) and score for internalizing and externalizing behaviors; and (vii) the full-scale IQ.

### Imputation of HLA variants and MHC haplotypes

HLA imputation was performed using the SNP2HLA software [39]. This bioinformatic tool allows researchers to impute from the chromosome 6 genotypic data of each patient, namely: (i) the HLA class I and class II-classical alleles at a 4-digit level; (ii) the amino acid variants from the imputed 4-digit HLA alleles; and (iii) the SNPs of the entire MHC region.

Specifically, we extracted 41,111 chromosome 6 variants from the chip genotype data and imputed a total of 8,961 HLA variants (424 classical HLA alleles and 1276 amino acid variations). We performed a second quality control on imputed markers, namely: the variants with minor allele frequency < 0.03 and/or *p* value for the Hardy–Weinberg departure test < 1 × 10^−6^ were removed (*N* = 1546). To ensure the best imputation quality, we applied a filter on the imputation score R2, and kept all variants with R2 > 0.8 with consequent removal of 12 additional variants. The resulting 7,403 variants, including 106 classical HLA alleles, 930 amino acid variations, and the 6367 SNPs were subjected to the statistical analysis pipeline.

As well as the single SNP effect, we also analyzed the potential impact of haplotypes involving the most significant SNPs. In addition, we looked for the possible effects of composite haplotypes involving the SNPs along the neighboring class II-classical HLA alleles genes, namely HLA-DQA1, HLA-DQB1, and HLA-DRB1. We also computed those having significant SNPs and suggestive HLA signals but failing to reach statistical significance. To this end, we used the package R HaploStats [50] to compute haplotypes. We obtained 508 haplotypes and as for the imputed variants, haplotypes with minor allele frequency < 0.03 were removed (*N* = 320) with PLINK [51].

### Statistical analysis

#### ASD vulnerability

We looked for genetic associations as to the risk to develop ASD among the 7403 imputed HLA variants and the 188 computed haplotypes. We compared ASD and TD for all the variants using a logistic regression implemented in PLINK software [51] under the additive, dominant, and recessive models. To correct for possible population stratification, we used the top four principal components (PC) as covariates in the statistical analyses. We also added gender and recruitment centers as covariates in our model. We used the Bonferroni method to correct for multiple testing. The statistical threshold of significance was set at 6.75 × 10^−6^, which was derived by dividing 0.05 by 7403 markers tested. This conservative threshold holds particular significance in our investigation due to the prominent presence of linkage disequilibrium (LD) in the MHC region. Through LD calculations, encompassing all variants in the region, we found a limited number of independent SNPs, i.e., 1349 (with a pruning based on a *r*^2^ value below 0.5). When considering the analysis for three different models (3 × 1349 = 4047 tests), the threshold became 1.24 × 10^−5^. However, for the sake of simplicity, we only retained the lowest threshold of 6.75 × 10^−6^ for all tests.

#### ASD severity

To explore if variants or haplotypes implicated in ASD risk can influence ASD severity, we used the psychometric tools assessed in the LEAP cohort (see above) to perform a multivariate linear regression using PLINK software [51] for each studied clinical scale under additive and dominant models only for the cases. To avoid spurious associations, we included age, IQ, sex, recruitment centers, and the first 4 PC as covariates for each model with the exception of IQ stratification. In the latter case, we used only age, sex, recruitment centers, and the first 4 PC as covariates in the model.

## Results

Initially, 1535 individuals (corresponding to 698 families) passed the genetic quality control procedures. After the selection of unrelated individuals from each family and stratification analysis (see Methods), the genotyping information from 528 unrelated individuals enrolled in the EU-AIMS/LEAP project (298 cases and 230 controls) were used for HLA imputation. Clinical and related information were available for 275 of the 298 cases. Socio-demographic characteristics are reported in Table 1 and showed a difference in the distribution according to gender, both in cases and in controls, where males were predominant (males: 71% for the ASD group and 63% for the controls). This distribution approached significance in comparison of cases and controls (*p* value = 0.053). Psychometric scores assessing clinical variables in ASD patients are reported in the supplementary Table S1.

**Table 1:** Socio-demographics information of all included individuals.

Characteristic	*N*	ASD, *N* = 298 mean (SD)	TD, *N* = 230 mean (SD)	*p* value
Age	482	16.90 (6.05)	17.70 (5.90)	0.14^a^
Sex	528			0.053^b^
F		84/298 (28%)	83/230 (36%)	
M		214/298 (72%)	147/230 (64%)	
Center	528			0.3^b^
Cambridge		43/298 (14%)	25/230 (11%)	
Karolinska		22/298 (7.4%)	21/230 (9.1%)	
KCL		87/298 (29%)	54/230 (23%)	
Mannheim		16/298 (5.4%)	20/230 (8.7%)	
Nijmegen		77/298 (26%)	56/230 (24%)	
Rome		18/298 (6.0%)	19/230 (8.3%)	
Utrecht		35/298 (12%)	35/230 (15%)	

^a^Welch two sample *t* test.

^b^Pearson’s chi-squared test.

### SNPs and HLA association analyses

**Fig. 1:**
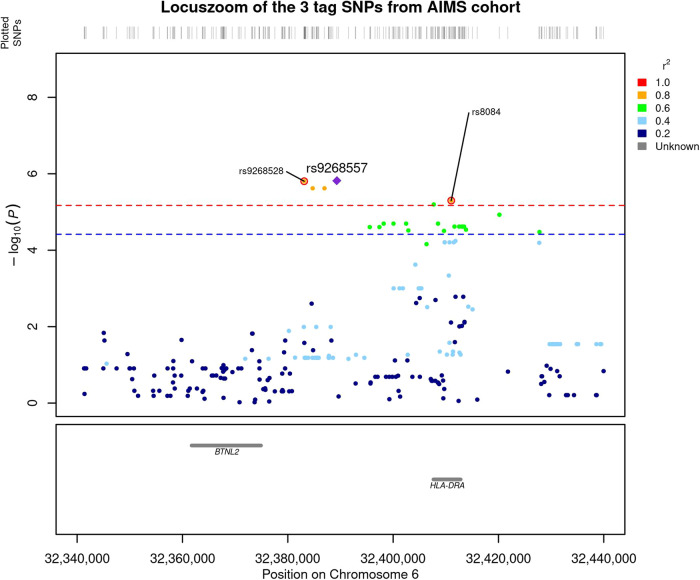
Locuszoom of the region containing the significant SNP in additive model. The –log_10_(*P* value of association) were plotted according to the physical position of the SNPs. The genes of the region are annotated at the bottom of the figure. The dashed line corresponds to the Bonferroni threshold. The colors of the SNPs indicate the LD with the strongest SNP rs9268557 measured by r^2^ coefficient. The SNPs tagging the three blocks are indicated.

**Fig. 2:**
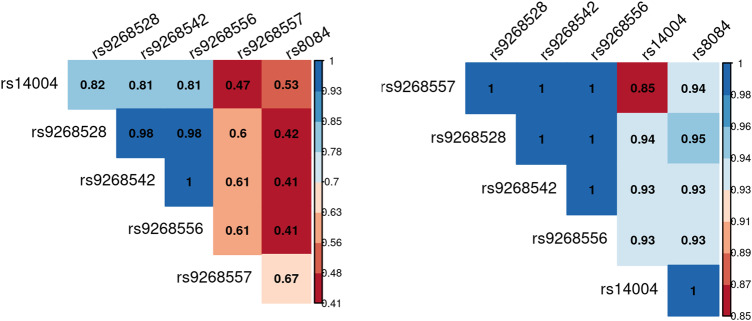
Linkage analysis of the 6 uncovered SNPs. The analysis of the linkage disequilibrium (LD) among the 6 SNPs shows three blocks of LD namely: (1) rs9268528, rs9268542, rs9268556, rs14004; (2) rs9268557 and (3) rs8084 which are not completely independent with an r2 greater than 0.4 and a D’ > 0.8.

We compared the genetic distribution of MHC SNPs, HLA alleles, and amino acids in 298 individuals with ASD and without ID, versus 230 non-autistic controls in additive, dominant and recessive models. While no classical HLA allele passed the Bonferroni threshold, six SNPs i.e., rs9268528, rs9268542, rs9268556, rs14004, rs9268557, and rs8084 were significantly associated with ASD risk in the additive model (Fig. 1 and Table 2). Three of them, namely rs9268528, rs9268542, and rs9268556, also passed the Bonferroni threshold in the dominant model (Table 2). By analyzing the linkage disequilibrium (LD) among the 6 SNPs, we obtained the three following blocks of LD (Fig. 2): (1) rs9268528, rs9268542, rs9268556, rs14004; (2) rs9268557 and (3) rs8084. However, these 3 blocks were not completely independent, with comparisons among them showing an r2 greater than 0.4 and the D’ > 0.8 (Fig. 2). Block 1 (*rs9268528-G, rs9268542-G*, *rs9268556-C,* and *rs14004-A*) associated with a protective effect against ASD risk, whilst the two remaining two blocks, namely the *rs9268557-T* and the *rs8084-A* alleles, associated with enhanced ASD risk (Table 2). In the control group, the allelic frequencies of such ASD-associated SNPs were in agreement with that of the 1000 genomes European population diversity. Although the allelic frequencies of the 6 SNPs were not always the same across centers, the distribution between cases and controls was always in the same direction (supplementary Table S2). As to location, the rs8084 and rs14004 mapped to the *HLA-DRA* gene [respectively at the junction between the second intron and the third exon and in the 5’-untranslated region (UTR)], whilst the four others were intergenic variants, located in a region encompassing the *BTNL2* (~10 kb) and *HLA-DRA* (~20 kb) loci (chr6: 32383108–32411035) (Fig. 1). Interestingly, beside these strongly associated variations, we also observed, in a recessive and a dominant manner respectively, two signals belonging to the HLA-DQB1 amino acids, namely a leucine at position 26 (DQB1-26-L) and a glycine at position 45 (DQB1-45–G) of the DQB1 heavy chain. While the two signals, although showing a strong association trend, failed initially to reach the established statistically significant threshold, we decided to investigate their possible implication in the modulation of ASD risk at haplotype level (see below).

**Table 2:** Association results of MHC region with ASD in additive model.

SNP	A1	A2	Freq A1 cases	Freq A1 controls	Additive model			Dominant model		
					OR	95% CI	*P*	OR	95% CI	*P*
rs9268557	T	C	57%	41%	1.90	[1.5; 2.5]	1.5 × 10^−6^	-	-	-
rs9268528	G	A	31%	46%	0.52	[0.4; 0.7]	1.6 × 10^−6^	0.4	[0.3; 0.6]	3.1 × 10^−6^
rs9268542	G	A	32%	47%	0.52	[0.4; 0.7]	2.4 × 10^−6^	0.4	[0.3; 0.6]	6 × 10^−6^
rs9268556	C	T	32%	47%	0.52	[0.4; 0.7]	2.4 × 10^−6^	0.4	[0.3; 0.6]	6 × 10^−6^
rs8084	A	C	49%	35%	1.86	[1.4; 2.4]	5.0 × 10^−6^	-	-	-
rs14004	A	C	34%	48%	0.55	[0.4; 0.7]	6.4 × 10^−6^	-	-	-

A1 is the tested allele and it has the minor allele frequency, *OR* odd ratio, *CI* confidence interval.

### Haplotype analyses

Since the 3 SNPs tagging the 3 blocks (*rs9268528-G*, *rs9268557-T,* and *rs8084-A*) are close to each other (Fig. 1), we investigated for possible haplotype effects. Moreover, as located within the HLA class II region, we also searched for haplotype effects between them and the classical class II HLA alleles (i.e. belonging to the *HLA-DQA1*, *DQB1,* and *DRB1* gene loci). Finally, we computed haplotypes involving the tag SNPs and the above-mentioned HLA-DQ beta-amino acids (DQB1-26-L and DQB1-45–G). We thus performed a logistic regression to compare the distribution of haplotypes in ASD patients and in controls with typical development, in the additive, dominant, and recessive models by means of the pipeline of analysis used for SNPs and HLA association analyses.

**Fig. 3:**
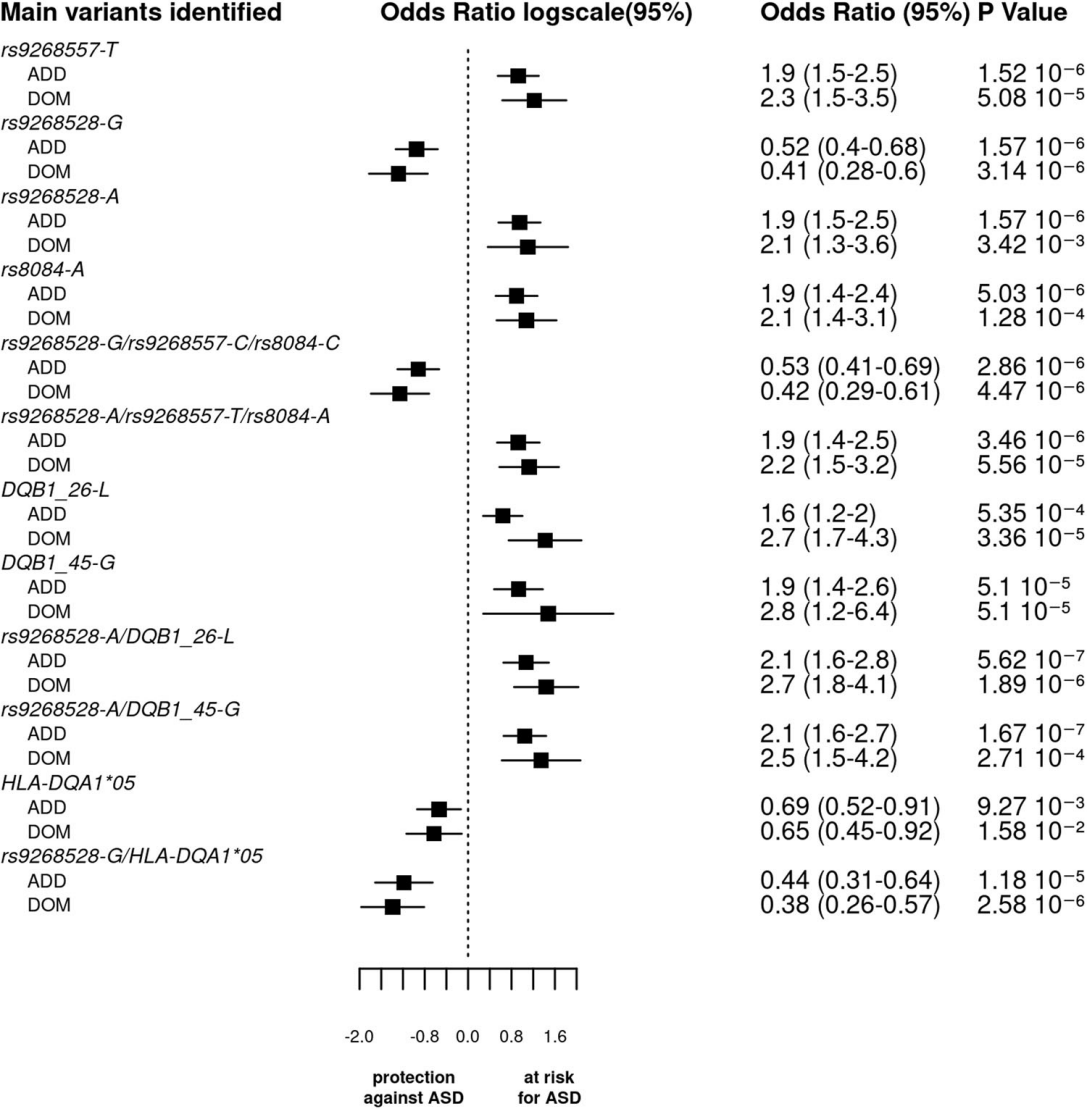
Logistic regression analysis of main genetic variants, a summary of Odds Ratios, and 95% Confidence Intervals. The bar graph displays the log odds ratios (OR) and their 95% confidence intervals (CI) for the main SNPs, haplotypes, and HLA (amino acid or classical alleles) based on the logistic regression analysis under both additive and dominant models. The vertical line represents the reference category. The horizontal bars represent the width of the 95% CI. The names of the variants are shown on the y-axis. The OR and *p* value are indicated on the right of the figure for each variant. As the p values of associations along odds ratio for the 3 tag SNPs haplotypes are comparable to that of the SNPs taken individually and since the 3 blocks of LD were not completely independent, we cannot conclude whether each variant by itself or their bearing haplotypes functionally drive ASD vulnerability.

Interestingly, in the additive model, haplotypes consisting of DQB1-26-L or DQB1-45-G amino acids and *rs9268528-A* or *rs9268557-T* were strongly associated with ASD risk. Indeed, the comparison of their distribution between ASD cases and TD subjects yielded lower p values than the most significant SNP in the additive model (*p* values: 1.66 × 10^−07^ and 1.5 × 10^−6^, respectively). Consequently, the odds ratios were also increased (2.1 for the most significant haplotype *vs* 1.90 for the *rs9268557-T* alone). This effect is even stronger in the dominant model for the haplotypes bearing the DQB1-26-L (Table 3 and Fig. 3).

**Table 3:** Main results of the haplotypes association analysis with ASD in additive, dominant and recessive model.

Variant and HLA gene or amino acid	Allele haplotype	Additive			Dominant			Recessive		
		OR	95% CI	*P*	OR	95% CI	*P*	OR	95% CI	*P*
rs9268528.AA_45_DQB1	A.G	2.07	[1.58–2.71]	**1.67** × **10**^**−7**^	-	-	-	2.53	[1.72–3.72]	2.15 × 10^−6^
rs9268557.AA_45_DQB1	T.G	2.03	[1.55–2.65]	**2.18** × **10**^**−7**^	2.58	[1.71–3.87]	5.20 × 10^−6^	2.43	[1.55–3.81]	1.13 × 10^−4^
rs9268557.AA_26_DQB1	T.L	2.04	[1.55–2.69]	**4.17** × **10**^**−7**^	2.67	[1.82–3.93]	**5.86** × **10**^**−7**^	2.17	[1.31–3.601]	2.62 × 10^−3^
rs9268528.AA_26_DQB1	A.L	2.10	[1.57–2.81]	**5.62** × **10**^**−7**^	2.71	[1.8–4.092]	1.89 × 10^−6^	2.21	[1.34–3.66]	2.03 × 10^−3^
rs9268528.rs9268557.rs8084.AA_45_DQB1	A.T.A.G	2.00	[1.52–2.64]	**8.82** × **10**^**−7**^	2.43	[1.66–3.54]	4.42 × 10^−6^	2.40	[1.41–4.08]	1.29 × 10^−3^
rs8084.AA_45_DQB1	A.G	2.00	[1.52–2.64]	**9.51** × **10**^**−7**^	2.32	[1.59–3.40]	1.50 × 10^−5^	2.58	[1.52–4.38]	4.58 × 10^−4^
rs9268528.rs9268557.rs8084.AA_26_DQB1	A.T.A.L	2.02	[1.52–2.7]	**1.40** **×** **10**^**−6**^	2.58	[1.77–3.75]	**7.10** × **10**^**−7**^	2.00	[1.14–3.51]	1.56 × 10^−2^
rs8084.AA_26_DQB1	A.L	2.00	[1.50–2.66]	1.99 × 10^−6^	2.53	[1.74–3.68]	**1.19** × **10**^**−6**^	2.00	[1.14–3.51]	1.56 × 10^−3^
rs9268528.rs9268557.rs8084	G.C.C	0.53	[0.41–0.69]	2.86 × 10^−6^	0.42	[0.29–0.61]	4.47 × 10^−6^	0.48	[0.29–0.80]	5.19 × 10^−3^
rs9268528.rs9268557.rs8084	A.T.A	1.90	[1.45–2.49]	3.46 × 10^−6^	2.18	[1.49–3.20]	5.56 × 10^−5^	2.46	[1.47–4.11]	5.96 × 10^−4^
rs9268528.HLA-DQA1	G.HLA-DQA1*05	0.44	[0.31–0.64]	1.18 × 10^−5^	0.38	[0.26–0.57]	2.59 × 10^−6^	-	-	-
rs8084.HLA-DQA1	C.HLA-DQA1*05	0.45	[0.32–0.65]	1.55 × 10^−5^	0.40	[0.27–0.60]	5.79 × 10^−6^	-	-	-
rs9268557.HLA-DQA1	C.HLA-DQA1*05	0.46	[0.32–0.66]	1.63 × 10^−5^	0.41	[0.28–0.61]	6.43 × 10^−6^	-	-	-
rs9268528.AA_26_DQB1	G.Y/G	0.54	[0.40–0.72]	3.86 × 10^−5^	0.48	[0.34–069]	7.30 × 10^−5^	0.41	[1.189–0.88]	2.19 × 10^−2^
rs8084.AA_45_DQB1	C.E	0.52	[0.37–0.72]	7.04 × 10^−5^	0.44	[0.30–0.64]	2.73 × 10^−5^	2.58	[1.52–4.38]	4.58 × 10^−4^
rs9268557.HLA-DQB1	T.HLA-DQB1*06	1.87	[1.36–2.56]	1.12 × 10^−4^	-	-	-	-	-	-
rs9268557.AA_45_DQB1_45	C.E	0.54	[0.39–0.74]	1.45 × 10^−4^	0.46	[0.32–0.68]	7.29 × 10^−5^	-	-	-
HLA-DQA1.HLA-DQB1	HLA-DQA1*05.HLA-DQB1*03	0.49	[0.34–0.71]	1.92 × 10^−4^	-	-	-	-	-	-
rs9268557.DQB1_C.HLA-DQB1	C.HLA-DQB1*03	0.58	[0.44–0.77]	2.01 × 10^−4^	-	-	-	-	-	-
rs9268528.rs9268557.rs8084.AA_26_DQB1	G.C.C.Y/G	0.57	[0.42–0.76]	2.03 × 10^−4^	2.58	[1.77–3.75]	7.10 × 10^−7^	2.00	[1.14–3.51]	1.56 × 10^−2^

*AA_XX_DQB1* amino acid at position XX in HLA-DQB1, *OR* odd ratio, *CI* confidence interval.

Bold values identify statistical significance.

Again, in the additive model, we observed that the two haplotypes, including the 3 tag SNPs (rs9268528, rs9268557, and rs8084) have two opposing effects: with (*rs9268528-G*, *rs9268557-C*, *rs8084-C*) affording protection against ASD, whilst (*rs9268528-A*, *rs9268557-T, rs8084-A*) associated with enhanced ASD risk (Table 3 and Fig. 3). As the p values of associations along odds ratio for the two haplotypes are comparable to that of the SNPs taken individually and since the 3 blocks of LD were not completely independent, we cannot conclude whether each variant by itself or their bearing haplotypes functionally drive ASD vulnerability.

Further analysis of correlations between combined SNPs and classical HLA class II alleles allowed the identification of the following haplotypes: (i) the HLA-DQA1*05 allele with *rs9268528-G* or *rs8084-C* or *rs9268557-C*, that confer a protective status against ASD; (ii) the HLA-DQB1*06 variant allele with *rs9268557-T* which associates with raised ASD risk; and (iii) the HLA-DQB1*03 allele either with the *rs9268557-C* or with the HLA-DQA1*05 variant both conferring a protective effect (Table 3).

In the dominant model, we observed a strong association between ASD risk and haplotypes bearing the amino acid DQB1-26-L variant, namely DQB1-26-L-*rs*9268557*-T*, DQB1-26-L-*rs*9268528-A, DQB1-26-L*-rs8084-A,* and DQB1-26-L- *rs*9268557*-T*-*rs*9268528-A-*rs*8084*-A*. The observed at-risk effect was found to be stronger than in the additive model, suggesting a dominant effect of these haplotypes. The *p* value and the odds ratio for the protective haplotype harboring both *rs9268528-G* and HLA-DQA1*05 were lower than in additive model, suggesting that the conferred protective status against ASD could be exerted in a dominant manner.

Finally, in the recessive model, the only haplotype yielding a *p* value comparable to that of the individual SNPs is the at-risk haplotype combining the HLA-DQB1-45-G variant with the *rs9268528-A* allele.

Overall, the observed haplotype-derived at-risk/protective effects strongly suggest a genetic interplay between the genetic diversity of MHC SNPs (*BTNL2)* and HLA class II alleles or amino acid variants.

### Functional analysis of the associated SNPs

Functionally, it is notable that the rs8084 variation creates a splice acceptor site leading to a 25 amino acid deletion of the HLA-DR alpha chain, which affects its molecular conformation and cellular localization [52].

Further screening of the RegulomeDB database [53], which reports information concerning the potential transcriptomic properties of genomic SNPs, indicated that 3 SNPs (rs9268528, rs14004, and rs8084) had a good RegulomeDB rank of 1 f (Supplementary Table S3). This makes their location within regulatory elements plausible and therefore their involvement in gene transcription, given that the probability to have a RegulomeDB score better than 1 f is less than 5% (data not shown). Thus, from the haplotype blocks only rs9268528 and rs14004 (block 1) and rs8084 (block 3) reached this 1 f score (Supplementary Table 2).

**Fig. 4:**
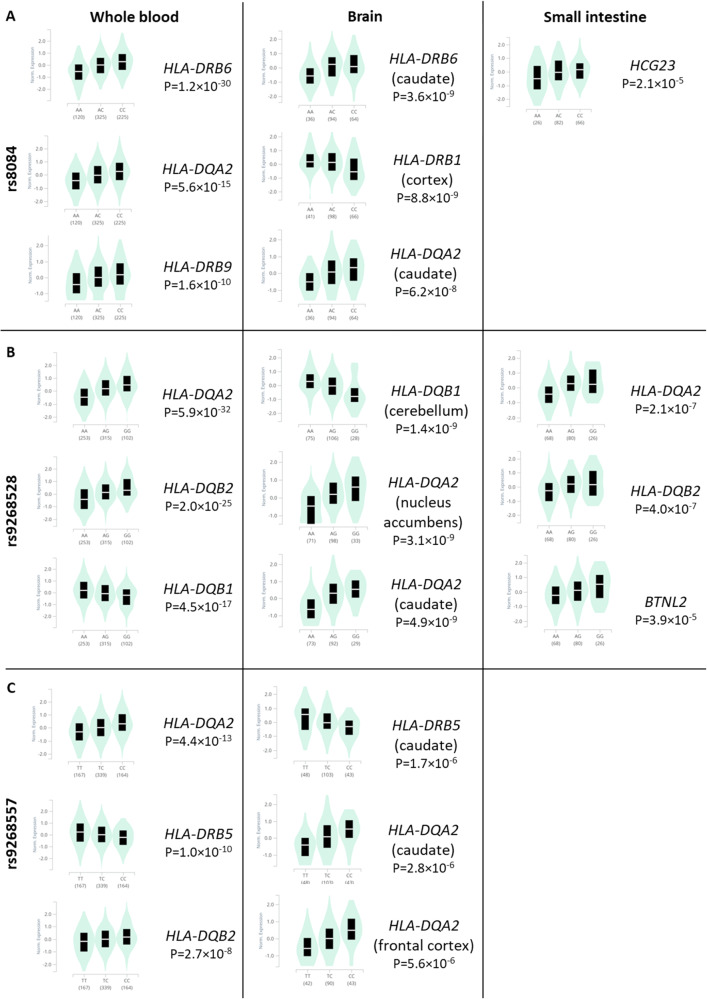
Graphical representation of the possible GTex derived quantitative trait loci (eQTL). Graphical representation of the possible GTex-derived quantitative trait loci (eQTL) linked to the SNPs rs8084 (**A**), rs9268528 (**B**), and rs9268557 (**C**), that tag the identified LD blocks in the brain, small intestine, and PBMCs. *P* values are more significant in whole blood, versus brain and small intestine. For all tissues and SNPs, we observed an overexpression of *HLA-DQA2*. The protective *rs9268528-G* allele is associated with the under-expression of the *DQB1* gene in the whole blood and brain. Conversely, *BTNL2*, well known for its involvement in inflammatory gastrointestinal disorders, is overexpressed in the small intestine in correlation with the protective allele *rs*9268528-G. HLA-DRB6 overexpression correlated with the *rs*8084-C variant in the whole blood and brain, whilst HLA-DBR5 was under-expressed when combined with the *rs9268557-C* variant in the same tissues.

We also used the GTex portal [54] to uncover possible quantitative trait loci (eQTL) linked to rs9268528, rs9268557, and rs8084 SNPs that tag the 3 identified blocks. We focused on three tissues known to be involved in ASD pathophysiology namely: brain, small intestine, and whole blood (PBMCs). Figure 4 depicts the most expressed genes and their correlations to these 3 SNPs in the brain, small intestine, and PBMCs. P values were more significant in whole blood, versus brain and small intestine. For all tissues and SNPs, we observed an overexpression of *HLA-DQA2*. Interestingly, the protective *rs9268528-G* allele associated with under-expression of the *HLA-DQB1* gene in the whole blood and brain (Fig. 4). Similarly, the *HLA-DQA1* gene is also under-expressed in the whole blood in correlation with the *rs9268528-G* allele (*p* = 10^−12^, beta = −0.15, not shown in Fig. 4). Conversely, *BTNL2*, well known for its involvement in inflammatory gastrointestinal disorders, was overexpressed in the small intestine in correlation with the protective allele *rs9268528-G*. Overall, these observations may reflect the above-mentioned genetic interplay between genetically determined low expression of the *HLA DQ* genes and high expression of *BTNL2* gene in conferring protection against ASD risk. Moreover, *HLA-DRB6* overexpression correlated with the *rs8084-C* variant in the whole blood and brain, whilst *HLA-DBR5* was under-expressed when combined with the *rs9268557-C* variant in the same tissues (Fig. 4). These observations reinforce the hypothesis that the observed tag SNPs may be having regulatory effects on HLA class II gene expression.

**Fig. 5:**
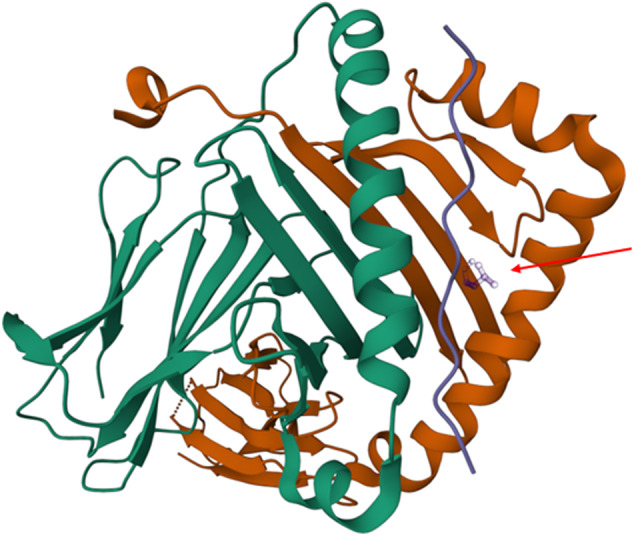
Graphical representation of the peptide binding groove of the HLA DQ heavy chain. The leucine at position 26 (DQB1-26L) which is located the pocket 4 is indicated by a red arrow. The DQB1-26L amino acid is shared by alleles belonging to the HLA-DQB1*02, HLA-DQB1* 03, and HLA-DQB1*06 specificities, whilst the DQB1-45G amino acid tag alleles of the HLA-DQB1*02, HLA-DQB1* 03, HLA-DQB1*04, HLA-DQB1* 05 and HLA-DQB1* 06 antigens.

Regarding the DQB1 amino acids, it is notable that the leucine at position 26 is in the peptide binding groove of the HLA DQ heavy chain and is part of the pocket 4 known to be pivotal for antigen presentation and repeatedly implicated as a risk factor for the development of ‘autoimmune’ disorders, including those targeting the gastrointestinal tract [55], (Fig. 5). The DQB1-26L amino acid is shared by alleles belonging to the HLA-DQB1*02, HLA-DQB1*03, and HLA-DQB1*06 specificities, whilst the DQB1-45G amino acid tag alleles of the HLA-DQB1*02, HLA-DQB1*03, HLA-DQB1*04, HLA-DQB1*05, and HLA-DQB1*06 antigens.

### SNP and haplotype influence on clinical characteristics in ASD patients

We then tested the potential influence of ASD-associated haplotypes and SNPs on ASD clinical presentation or severity for patients with available clinical annotations (*N* = 275). We, therefore, performed linear regression involving different psychometric tools assessed in the LEAP cohort, these scores were used to evaluate ASD characteristics and cognitive performance. Only trends toward association were observed between the genetic variants and the studied clinical scales (data not shown), highlighting the difficulties in comparing genetic traits with clinical symptoms that fluctuate over the course of a disorder.

## Discussion

Mutational events affecting synaptic and neuronal processes only explain a relatively small proportion of ASD cases [3, 4]. While genomic linkage scan and cytogenetic analysis allowed the first descriptions of at-risk gene alterations scattered across the human genome [56, 57], further GWAS expand such genetic diversity by identifying causal copy number variations (CNVs) including large recurrent deletions or duplications disrupting either single gene or a chromosomal region containing multiple genes [57]. Although such mutational events affect important candidate genes, they remain relatively rare explaining around 1% of ASD cases [3, 4]. Besides, common variants were also reported, especially in a recent very large GWAS that identify 88 variants significatively associated with ASD [58]. In this context, loci or genetic variants regulating immune processes have emerged as a cutting-edge area of research in ASD [25]. Various aspects of immune dysfunction underpin ASD pathophysiology, including overexpression of pro-inflammatory molecules, ‘autoimmune’ processes, and gastrointestinal dysbiosis [59]. Such immune dysregulation implicates the human leukocyte antigen (HLA) system, which is an important aspect of both innate and adaptive immunity. However, partly as a consequence of its complexity, the HLA system has been under-investigated in ASD [23–25].

After imputation of the MHC/HLA variants from individuals included in a GWAS study, we analyzed their potential associations with ASD and identified six variants crossing the Bonferroni threshold. Four variants (rs9268528, rs9268542, rs9268556, and rs9268557) are located in close proximity to the MHC class II gene-linked butyrophilin-like 2 (*BTNL2*) locus. *BTNL2* belongs to the B7 superfamily, which are crucial regulators of T-cell activation and tolerance (for review see Nguyen et al., 2006; Arnett & Viney, 2014 [60, 61]). BTNL2 is highly expressed both in the lymphoid tissues and in the intestine. BTNL2 inhibits T-cell proliferation, thereby linking *BTNL2* polymorphisms with inflammatory/’autoimmune’ diseases such as sarcoidosis or myositis [62–64]. BTNL2 expression in lymphoid organs indicates a role in initiating immune responses, whilst the abundant expression of *BTNL2* mRNA in the intestine indicates a pivotal role in the modulation of mucosal immunity and/or tolerance. Within the GI-tract, *BTNL2* is a significant homeostatic regulator of tissue-resident gamma delta (γδ) intraepithelial lymphocytes (IELs). γδ IELs are a heterogeneous population of immune cells in the intestinal epithelium, which are crucial to the maintenance of gut homeostasis [65]. γδ IELs can strengthen tight junctions and orchestrate innate and adaptive immunity during homeostasis, inflammation, and infection, thereby having an important role in the regulation of intestinal epithelial barrier integrity [65]. BTNL2 acts to suppress γδ IELs proliferation, preferentially in the ileum, as demonstrated by transcriptomic characterization of murine site-specific BTNL2-KO γδ IELs, which showed BTNL2 to regulate the antimicrobial response of ileal γδ IELs [66]. Dextran sulfate sodium (DSS)-induced colitis in BTNL2-KO mice enhances inflammation and delays mucosal repair in the colon, highlighting the role of BTNL2 in regulating inflammatory signals. Since ileal γδ IEL motility along the villi-crypt axis is strictly dependent on the presence of microbiota [66], gut dysbiosis and an altered antibacterial profile may contribute to improper localization and/or ineffective surveillance by ileal BTNL2-KO γδ IELs. Suppressed BTNL2 levels may therefore contribute to the initiation and/or maintenance of inflammation in the ileum, with consequences for ASD pathophysiology and behaviors. This is supported by data from the GTex database showing *BTNL2* to be overexpressed in the small intestine in correlation with the *rs9268528-G* SNP allele. This is likely to be the underpinnings of the protection against ASD afforded by this SNP and by the protective block 1 haplotype that carries it.

The gut-brain axis is intimately associated with many neuro-psychiatric and neurodevelopmental disorders, including ASD [67, 68]. This has relevance to the digestive symptoms commonly observed in a substantial subset of autistic people, which will often be concurrent to alterations in the gut microbiome in ASD [7], and supported by a recent meta-analysis [69]. However, in a study of pre-pubertal Chinese patients with ASD, gut microbiota alterations seem independent of comorbid functional gastrointestinal disorders, perhaps indicative of distinct genetic control of the observed dysbiosis in ASD patients [70].

The second top signal also comes from the HLA-class II region and consists of two SNPs (rs8084 and rs14004) both belonging to the *HLA-DRA* gene, which encodes the non-variable HLA-DR alpha subunit that can associate with any hypervariable HLA-DR beta chain to form mature HLA-DRB molecules. rs8084 creates a cryptic splice site in the *DRA* mRNA and is associated with immune conditions including psoriasis [71] and allergy to penicillin [72], whilst the 5-UTR rs14004 modulates both *HLA-DR* and *DQ* gene expression and may increase type 1 diabetes mellitus risk [73]. These two variations exhibit a good RegulomeDB rank, substantiating their involvement in transcriptomic processes. Accordingly, the *rs8084-C* SNP allele belongs to the protective *rs9268528-G, rs9268557-C, rs8084-C* haplotype while its *rs8084-A* counterpart allele is a part of the *rs9268528-A, rs9268557-T, rs8084-A* as a risk haplotype. Given the monomorphic status of the HLA-DRA molecule, it is likely that the uncovered association may reflect either the functional relevance of the two SNPs per se or possible linkage disequilibrium with other loci in the HLA class II region (Fig. 1).

The strongest signals in the current investigation are derived from the haplotype combinations between the DQB1-26-L and DQB1-45-G amino acids and the two SNPs tagging the *BTNL2* region, namely rs9268528 and rs9268557. GTex portal screening showed *DQA1* and *DQB1* genes to be overexpressed in whole blood in correlation with the at-risk allele*, rs9268528-A*. This likely indicates an interaction between *BTNL2* and *HLA-DQ* loci that increases ASD risk, whereby lower BTNL2 expression, coupled with HLA-DQA1 and HLA-DQB1 overexpression will exacerbate inflammation, perhaps especially in the GI-tract given the role of *BTNL2* in GI-tract homeostasis. It is proposed that dysregulated intestinal inflammation will underpin that heightened ASD vulnerability in carriers of these haplotypes. Similarly, haplotypes carriers of the at-risk allele *rs9268528-A* and one of the two above-mentioned amino acids (leucine-26 and glycine-45) will also have an increased ASD risk. These two amino acids tag specifically the following HLA alleles: HLA-DQB1*02 (DQ2), HLA-DQB1*03:02 (DQ8), HLA-DQB1*04, HLA-DQB1*05 and HLA-DQB1*06, which have mostly been associated with ‘autoimmune’ and inflammatory disorders, including celiac disease, allergy to hydrolyzed wheat proteins, autoimmune pancreatitis, inflammatory bowel disease, and narcolepsy [55, 74–78]. This may be of particular relevance in celiac disease, where the HLA-DQB1*02 and HLA-DQB1*03:02 alleles are pivotal [74].

In line with the role of GI-tract inflammation in ASD risk, the dominant model also showed a protective status conferred by the composite haplotype HLA-DQA1*05 allele rs9268528*-G*. Such observations reinforces the parallel with celiac disease [74], where the most associated HLA allele is the HLA-DQ2 antigenic specificity, which consists of a molecular heterodimer encoded by the HLA-DQA1*05 and the HLA-DQB1*02 alleles. HLA-DQA1*05 has been associated with GI-tract inflammation for many years, including raised symptom severity in ulcerative colitis (UC) and Crohn’s disease at diagnosis, in association with a more rapid onset, and an earlier need for immunosuppressive or biological treatment [79]. The HLA-DQA1*05 allele is also associated with anti-drug antibody production against biotherapies in Crohn’s disease patients, further contributing to its pro-inflammatory properties [80]. Consequently, due to its involvement in GI-tract inflammatory and allergic processes, both of which are aspects of ASD pathophysiology [5, 81], the HLA-DQA1*0501 allele may be of particular relevance to ASD pathoetiology. The haplotypic analysis also highlight the possible involvement of other HLA alleles, namely HLA-DQB1*03, and HLA-DQB1*06, which have all previously been associated with celiac disease risk.

Overall, this investigation highlights an interplay between two distinct but intertwined genetic elements, namely *BTNL2* and the HLA class II, which may underpin ASD risk. As well as the modulation of T-cell-related properties, BTNL2 may also modulate HLA class II allele expression, with consequences for immune/’autoimmune’ disorders. The present findings agree with and extend previous observations concerning the genetic association between ASD and variants related to GI tract homeostasis. In consecutive studies, we showed that: (i) the *CLEC7A* gene, which encodes the fungal sensor, Dectin-1, also modulate the risk of developing severe ulcerative colitis [82], and acts as a genetic disease specifier of cognition in high-functioning ASD [83]; (ii) an HLA haplotype previously associated with pediatric celiac disease, HLA-DRB1*11-HLA-DQB1*07, increase ASD risk, associating with higher social and non-verbal capacity [23]; and (iii) an HLA-class II sub-haplotype, which modulates regressive ASD risk, an autistic endophenotype characterized by intestinal anatomical alterations [24]. Notably, the MET receptor tyrosine kinase gene that encode molecules involved in gastrointestinal repair, immune function, neocortical, and cerebellar growth [84] was also repeatedly associated with ASD risk by independent studies [85]. The gastrointestinal component of ASD clearly has an immunogenetic aspect, relevant to both innate and adaptive immune processes. Gut microbiota diversity seems importantly determined by HLA diversity, as indicated by data on a large general population sample by Andeweg and colleagues showing HLA haplotypes to associate with gut microbiome composition [28]. Notably, the authors did not exclude the involvement of the non-conventional MHC class I-related gene A (*MICA*) and *MICB* loci, which are the main modulators of resident γδ IELs. γδ T cells represent a small population of T cells, being distinct from α/β TCR. γδ IELs are a subset of γδ T cells around the gut being concentrated in the intestinal mucosa and appear to have a prominent role in recognizing small bacterial phosphoantigens and undefined antigens presented by MICA/MICB proteins. γδ T cells have potent cytotoxic activity, being an important link between innate and adaptive immunity. γδ T cells may be especially important in the first 6 months postnatally, when the adaptive immune system is still developing, with this thought to be a crucial time in the pathoetiology of ASD. The role of early developmental processes is also highlighted by the association of the HLA-DQ2 genotype with the early developmental pathoetiology of celiac disease, with the HLA-DQ2 genotype associating with gut microbiota composition [86]. Finally, a pathophysiological link between the γδ IELs and the HLA-DQA1*0501 allele may be evident in a study analyzing the cytotoxic properties of PBMCs, which showed specific recognition of cells carrying the DQA1*0501 by lymphocytes bearing γδ TCR [87].

The data above indicate that genetically deficient BTNL2, coupled with high expression of associated HLA alleles can create favorable conditions for the initiation, maintenance, or exacerbation of inflammation in the ileum with downstream inflammatory processes possibly impacting ASD risk and/or symptom severity. Diminished expression of BTNL2 may dysregulate ileal γδ IELs, with consequent intestinal inflammation, whilst overexpression of the celiac disease-associated HLA-DQA1*05 or other pro-inflammatory HLA alleles will further contribute to inflammation. This study is the first to identify a genetic link between the GI tract and ASD.

It is important to note that the herein uncovered genetic variations were not previously identified in other Genome-Wide Association Studies, including a recent and extensive one conducted by Grove et al. [58]. This discrepancy may be explained by the heterogeneity of ASD, as pointed out by Warrier et al. [88, 89], which can arise from variations in diagnostic criteria and associated features such as IQ, adaptive behavior, and motor coordination. Our study focused specifically on ASD without intellectual disability, whereas Grove et al. [58] included patients with various types of ASD including Pervasive Developmental Disorder (PDD) and PDD-Not Otherwise Specified (PDD-NOS), atypical autism with or without ID, as well as some controls with ID. In our study, we specifically focused on ASD without intellectual disability, whereas Grove et al. [50] included patients with various types of ASD, such as Pervasive Developmental Disorder (PDD), PDD-Not Otherwise Specified (PDD-NOS), atypical autism with or without intellectual disability, as well as controls with intellectual disability. Furthermore, we rigorously selected participants of European descent from various countries, while Grove et al. [58] included a mix of populations from Denmark, North America, Europe, and Canada, which could affect the results of HLA analysis given the well-known anthropogenetic distribution of HLA alleles. Additionally, our study was performed under a classical case-control design, while Grove et al. conducted a meta-analysis of results from a case-control study (iPSYCH) and PGC family-based results obtained using pseudo-controls. Additionally, our study was performed under a classical case-control design, whereas Grove et al. conducted a meta-analysis of results from a case-control study (iPSYCH) and family-based results obtained using pseudo-controls from the Psychiatric Genomics Consortium (PGC). These differences in study design may contribute to the apparent inconsistency in the detection of genetic variations in the MHC/HLA region, which has been previously linked to ASD by the Cross-Disorder Group of the Psychiatric Genomics Consortium (2013) [21]. Such possibility emphasizes the critical importance of carefully considering the study design as well as patient population selection when investigating the genetic underpinnings of complex disorders like ASD, particularly within complex clusters such as the MHC/HLA. Another important aspect is the herein exclusive focus on the major histocompatibility complex/human leukocyte antigen (MHC/HLA) region, using a similar approach as that of the Nudel study [22]. In contrast to Nudel study [22], our investigation encompassed not only the classical HLA variants but also individual single nucleotide polymorphisms (SNPs) and amino acid variants within the MHC region, which may explain the new results we observed. The strongest signal rs9268757 comes with an OR reaching 1.9, which is quite high for a GWAS. We acknowledge that the population studied is relatively small (a few hundred patients), and it is unlikely that such a high OR will be obtained in a replication study. Nevertheless, in spite of the variability of the MHC allele distribution between populations, we have been able to check that the distribution of the main SNP alleles identified was similar and in the same direction in the four main European centers included in the study (with at least 20 cases and controls per center, see supp Table 2). Thus, these significant signals are good candidates, and they already provide an interesting biological interpretation for the molecular etiology of autism without ID, which is indeed the primary goal of a GWAS. Given the preliminary nature of our work with a globally relatively small sample size, further confirmation and replication of the findings are warranted before translation to clinical applications.

Some limitations deserve to be mentioned: (i) the non-availability of data concerning either history/stigma of infections or comorbid somatic symptoms, including those related to the gastrointestinal tract, (ii) the non-ability of creating additional study group with IQ greater than 85 as IQs between 75 and 85 may corresponds to patients with some intellectual impairments [90] and (iii) missing data pertaining to severity possibly explaining the absence of associations between the uncovered variations and the severity of ASD even if it is also possible that the genetic factors influencing susceptibility and those influencing the severity of autism may not necessarily coincide or overlap.

In summary, BTNL2, γδ IELs, and HLA class II variants and interactions may be an important mediator of how the gut-brain axis links to ASD pathoetiology and pathophysiology. Future studies will be required to replicate and extend the data above, including exploring how such susceptibility alleles may shape gut-targeted therapies.

## Supplementary information

Supplementary Figure 1

S1

S2

S3

## Data Availability

All the data generated in the present study are fully available.
