# Supplementary material for: A human leukocyte antigen imputation study uncovers possible genetic interplay between gut inflammatory processes and autism spectrum disorders
Source: Transl Psychiatry. 2023 Jul 6;13:244. doi: 10.1038/s41398-023-02550-y (PMC10322870; doi:10.1038/s41398-023-02550-y)
Supplement: Supplementary file 1 — Supplementary Figure 1 [file 41398_2023_2550_MOESM1_ESM.pdf]

Supplementary Figure 1

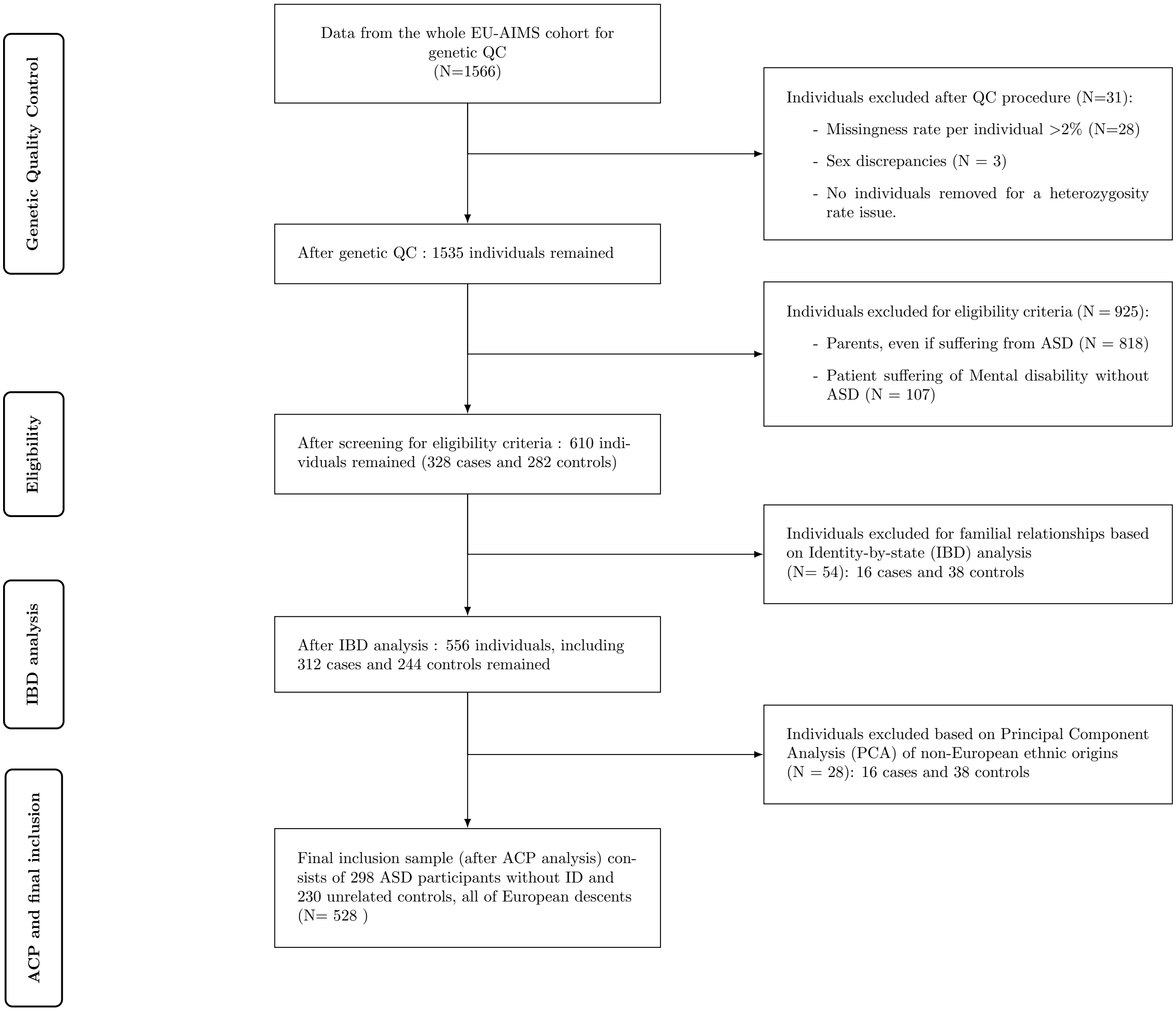

Flowchart illustrating the process of patient selection for inclusion in our analysis. The flowchart outlines the step-by-step procedure for the recruitment and inclusion of participants in the genetic study.
