# Supplementary material for: A human leukocyte antigen imputation study uncovers possible genetic interplay between gut inflammatory processes and autism spectrum disorders
Source: Transl Psychiatry. 2023 Jul 6;13:244. doi: 10.1038/s41398-023-02550-y (PMC10322870; doi:10.1038/s41398-023-02550-y)
Supplement: Supplementary file 2 — S1 [file 41398_2023_2550_MOESM2_ESM.docx]

**Table S1: Clinical information of ASD patients**

| Characteristic | N | ASD, N = 298^1^ |
| --- | --- | --- |
| **Full-scale IQ** | 273 | 103.76 (15.52) |
| Not Provided |  | 25 |
| **ADI-Social** | 266 | 15.79 (6.72) |
| Not Provided |  | 32 |
| **ADI-Communication** | 266 | 12.86 (5.55) |
| Not Provided |  | 32 |
| **ADI-Rrb** | 266 | 4.24 (2.63) |
| Not Provided |  | 32 |
| **ADOS-Total** | 268 | 5.29 (2.72) |
| Not Provided |  | 30 |
| **ADOS-Social** | 268 | 5.96 (2.55) |
| Not Provided |  | 30 |
| **ADOS-****Rrb** | 268 | 4.84 (2.67) |
| Not Provided |  | 30 |
| **SRS-T-score** | 248 | 69.53 (12.17) |
| Not Provided |  | 50 |
| **SSP** | 160 | 140.51 (27.30) |
| Not Provided |  | 138 |
| **SDQ-Externalizing Score** | 216 | 7.67 (4.00) |
| Not Provided |  | 82 |
|  |  |  |
| Characteristic | N | ASD, N = 298^2^ |
| **DAWBA-Depression** | 227 |  |
| 0 |  | 112 / 227 (49%) |
| 1 |  | 76 / 227 (33%) |
| 2 |  | 8 / 227 (3.5%) |
| 3 |  | 20 / 227 (8.8%) |
| 4 |  | 8 / 227 (3.5%) |
| 5 |  | 3 / 227 (1.3%) |
| Not Provided |  | 71 |
| **DAWBA-ADHD** | 198 |  |
| 0 |  | 64 / 198 (32%) |
| 1 |  | 22 / 198 (11%) |
| 2 |  | 24 / 198 (12%) |
| 3 |  | 55 / 198 (28%) |
| 4 |  | 27 / 198 (14%) |
| Characteristic | N | ASD, N = 298^2^ |
| 5 |  | 6 / 198 (3.0%) |
| Not Provided |  | 100 |
| **DAWBA-Anxiety Disorders** | 245 |  |
| 0 |  | 8 / 245 (3.3%) |
| 1 |  | 57 / 245 (23%) |
| 2 |  | 54 / 245 (22%) |
| 3 |  | 53 / 245 (22%) |
| 4 |  | 60 / 245 (24%) |
| 5 |  | 13 / 245 (5.3%) |
| Not Provided |  | 53 |
| **DAWBA-Behavioral Disorder** | 129 |  |
| 1 |  | 23 / 129 (18%) |
| 2 |  | 45 / 129 (35%) |
| 3 |  | 22 / 129 (17%) |
| 4 |  | 31 / 129 (24%) |
| 5 |  | 8 / 129 (6.2%) |
| Not Provided |  | 169 |
| **DAWBA-Internalizing Behavior** | 245 |  |
| 0 |  | 8 / 245 (3.3%) |
| 1 |  | 54 / 245 (22%) |
| 2 |  | 49 / 245 (20%) |
| 3 |  | 56 / 245 (23%) |
| 4 |  | 62 / 245 (25%) |
| 5 |  | 16 / 245 (6.5%) |
| Not Provided |  | 53 |
| **DAWBA-Externalizing Behavior** | 245 |  |
| 0 |  | 81 / 245 (33%) |
| 1 |  | 27 / 245 (11%) |
| 2 |  | 34 / 245 (14%) |
| 3 |  | 45 / 245 (18%) |
| 4 |  | 45 / 245 (18%) |
| 5 |  | 13 / 245 (5.3%) |
| Not Provided |  | 53 |
| ^1^Mean (SD); ^2^n / N (%) | | |

IQ, Intelligence quotient; ADI, Autism Diagnostic Interview; ADOS, Autism Diagnostic Observation Schedule; Rrb, restricted and repetitive behaviours; SRS, Social Responsiveness Scale; SSP, Short Sensory Profile; SDQ, the Strength and Difficulties Questionnaire; DAWBA, Development and Well-Being Assessment; ADHD, Attention Deficit - Hyperactivity disorders.
