# Supplementary material for: A human leukocyte antigen imputation study uncovers possible genetic interplay between gut inflammatory processes and autism spectrum disorders
Source: Transl Psychiatry. 2023 Jul 6;13:244. doi: 10.1038/s41398-023-02550-y (PMC10322870; doi:10.1038/s41398-023-02550-y)
Supplement: Supplementary file 3 — S2 [file 41398_2023_2550_MOESM3_ESM.docx]

**Table S2: Frequency of Tag SNPs in different recruitment centers**

This table provides reference data from the 1000 Genomes (1000G) project for the European population. This reference information includes the minimum and maximum frequencies of the tag SNPs observed in the European population. Additionally, the table displays the frequency of the tag SNP in our study cohort as well as in the four major recruitment centers. The table also presents the population sizes (effectives) for each population in our cohort, excluding the 1000G reference population. All frequencies are reported as percentages.

|  |  | 1000G | LEAP | | UK/Cambridge^1^ | | Netherlands/Utrecht^2^ | | Netherlands/Nijmegen^3^ | | UK/London^4^ | |
| --- | --- | --- | --- | --- | --- | --- | --- | --- | --- | --- | --- | --- |
| SNPs Name | Tested allele | European  [min-max] | Controls  (N=230) | Cases  (N=298) | Controls  (N=25) | Cases  (N=43) | Controls  (N=35) | Cases  (N=35) | Controls  (N=56) | Cases  (N=77) | Controls  (N=54) | Cases  (N=87) |
| rs9268557 | T | 50 [48-54] | 41 | 57 | 34 | 58,14 | 40 | 60 | 49,11 | 58,44 | 49,07 | 57,47 |
| rs9268528 | G | 37 [30-43] | 46 | 31 | 54 | 25,58 | 48,57 | 25,71 | 42,86 | 29,87 | 42,59 | 32,18 |
| rs8084 | A | 41 [35-49] | 35 | 49 | 30 | 50 | 34,29 | 57,14 | 46,43 | 54,55 | 41,67 | 44,83 |

^1^ Autism Research Centre, University of Cambridge (UCAM, UK); ^2^ University Medical Centre Utrecht (UMCU, Netherlands), ^3^ Radboud University Medical Centre (RUMC, Netherlands); ^4^. Institute of Psychiatry, Psychology and Neuroscience, King’s College London (IoPPN/KCL, UK)
