# Supplementary material for: A human leukocyte antigen imputation study uncovers possible genetic interplay between gut inflammatory processes and autism spectrum disorders
Source: Transl Psychiatry. 2023 Jul 6;13:244. doi: 10.1038/s41398-023-02550-y (PMC10322870; doi:10.1038/s41398-023-02550-y)
Supplement: Supplementary file 4 — S3 [file 41398_2023_2550_MOESM4_ESM.docx]

**Table S3: Summary of information about the significant SNPs associated with ASD phenotype in MHC region.**

| rsID | ref | alt | freq alt EUR | Proximity gene | Distances (in kb) from BTNL2 | Distances (in kb) from HLA_DRA | RegulomeDB rank |
| --- | --- | --- | --- | --- | --- | --- | --- |
| rs9268528 | A | G | 37% | BTNL2 | 8203 | 24511 | 1f |
| rs9268542 | A | G | 38% | BTNL2 | 9816 | 22898 | 7 |
| rs9268556 | T | C | 38% | BTNL2 | 12059 | 20655 | 6 |
| rs9268557 | T | C | 50% | BTNL2 | 14400 | 18314 | 6 |
| rs14004 | C | A | 38% | HLA-DRA | 32804 | . | 1f |
| rs8084 | A | C | 56% | HLA-DRA | 36130 | . | 1f |
